# Enhanced YOLOv11 Framework for Accurate Multi-Fault Detection in UAV Photovoltaic Inspection

**DOI:** 10.3390/s25175311

**Published:** 2025-08-26

**Authors:** Shufeng Meng, Yang Yue, Tianxu Xu

**Affiliations:** 1 School of Electrical and Information Engineering, Zhengzhou University, Zhengzhou 450001, China; shufengmeng@stu.zzu.edu.cn; 2 School of Information and Communications Engineering, Xi’an Jiaotong University, Xi’an 710049, China; yueyang@xjtu.edu.cn

**Keywords:** photovoltaic fault detection, YOLOv11, HSV color model, outlook attention, mix structure block, Grad-CAM

## Abstract

Stains, defects, and snow accumulation constitute three prevalent photovoltaic (PV) anomalies; each exhibits unique color and thermal signatures yet collectively curtail energy yield. Existing detectors typically sacrifice accuracy for speed, and none simultaneously classify all three fault types. To counter the identified limitations, an enhanced YOLOv11 framework is introduced. First, the hue-saturation-value (HSV) color model is employed to decouple hue and brightness, strengthening color feature extraction and cross-sensor generalization. Second, an outlook attention module integrated into the backbone precisely delineates micro-defect boundaries. Third, a mix structure block in the detection head encodes global context and fine-grained details to boost small object recognition. Additionally, the bounded sigmoid linear unit (B-SiLU) activation function optimizes gradient flow and feature discrimination through an improved nonlinear mapping, while the gradient-weighted class activation mapping (Grad-CAM) visualizations confirm selective attention to fault regions. Experimental results show that overall mean average precision (mAP) rises by 1.8%, with defect, stain, and snow accuracies improving by 2.2%, 3.3%, and 0.8%, respectively, offering a reliable solution for intelligent PV inspection and early fault detection.

## 1. Introduction

Amid the accelerating shift toward a decarbonized energy landscape, photovoltaic (PV) arrays have become indispensable pillars of sustainable development and strategic levers for achieving carbon neutrality, renowned for their carbon-free and renewable attributes [1]. However, as these modules proliferate across deserts, plateaus, and snow belts, they face exposure to extreme climates and topographically complex sites, jeopardizing their long-term reliability [2]. External contaminants such as avian excreta, mineral dust, and industrial aerosols scatter or absorb incident irradiance, eroding optical transmittance and consequently diminishing conversion efficiency [3,4]. Concurrently, latent internal anomalies like hotspot clusters and micro-crack networks reduce active cell area, intensify resistive heating, and heighten the probability of catastrophic thermal runaway [5,6]. Seasonal snow further compounds these losses by masking entire panels, curtailing photon capture and precipitating steep declines in electricity yield [7,8,9]. Therefore, confronting these interrelated degradation pathways demands an integrated diagnostic strategy capable of concurrently sensing surface soiling, internal impairment, and snow occlusion. Such a unified monitoring framework is essential to safeguard PV performance, extend asset lifetimes, and maximize energy harvest across diverse operating environments.

Traditional fault detection in PV modules primarily relies on human inspection and basic image analysis techniques. Such methods are characterized by low efficiency, a strong dependence on human experience, and difficulties in scaling up to meet the operational and maintenance requirements of large-scale PV systems [10,11]. Amidst the swift advancement of artificial intelligence and visual analytics, intelligent detection methods leveraging visual pattern recognition have progressively established themselves as a significant research focus and have been broadly implemented within the domain of PV anomaly detection. Within the context of stain detection, numerous studies have been dedicated to refining model architectures to enhance detection efficacy and streamline model parameters. A case in point is the research by Liu et al. [12], who devised an object detection framework centered around deep neural networks that integrates transfer learning, convolutional attention modules, and deformable detection transformers. This model has increased the detection accuracy of small target stains, such as leaves and bird droppings, by 6.6%, but exhibited limitations such as low frame rate (FPS) and high latency. Umair Naeem et al. [13] developed a customized model that integrates a convolutional block attention module and two specialized detection heads optimized for dust and bird droppings. This model achieved a significant improvement in mean average precision at 50% intersection over union (mAP@50) for bird droppings and dust by 40.2%, while reducing the number of parameters by 24%. Gao et al. [14] developed a segmentation technique based on multi-channel fusion, which accurately identifies bird droppings on the surface of photovoltaic modules and embeds the segmentation results into the input feature space to augment the performance of traditional mask region convolutional neural networks (R-CNN). This approach achieved an increase of 5.9% in mean average precision (mAP) for object detection.

Recent efforts toward internal defect localization have pursued architectural ingenuity and learning strategy refinement. Wang et al. [15] embedded a channel-offset transformation (COT) self-attention unit and redesigned the spatial pyramid pooling and cross stage partial channel (SPPCSPC) backbone of YOLOv7, boosting small-target sensitivity in cluttered scenes with a detection accuracy of 98.08%. Feng et al. [16] introduced a reversible column bottleneck and an efficient multi-scale attention (EMA) mechanism, maintaining prediction fidelity while reducing model complexity. Han et al. [17] grafted lightweight group shuffle convolution (GSConv) layers and a multi-level feature selective fusion (MLFSF) component into a real-time detection transformer (RT-DETR), achieving a 3.9% accuracy gain alongside an 11.83% parameter reduction. Kang et al. [18] leveraged weak supervision to train a CNN for PV cell inspection, alleviating annotation burdens and enhancing simultaneous detection and classification of diverse defect morphologies.

In snow-related diagnostics, Baldus-Juersen et al. [19] devised image analytic routines that quantify snow cover on PV arrays and couple them with energy loss models, attaining 90% detection precision. Mari B. Øgaard et al. [8] extracted snow specific sig-natures from PV data, enhancing snowfall models, and correctly identifying 97% of recorded snow events. Collectively, these studies advance accuracy, throughput, or model compactness for stains, defects, and snow. However, despite significant progress in individual anomaly detection, current methodologies predominantly address specific types of anomalies in isolation, neglecting the need for a unified framework capable of simultaneously addressing multiple anomalies. This limitation is particularly evident in extensive photovoltaic systems, where diverse anomalies often coexist, thereby necessitating a comprehensive and integrated detection approach.

Among contemporary detection paradigms, the YOLO family has emerged as the de-facto choice for PV fault inspection owing to its fully-convolutional, single-shot formulation that yields end-to-end latency in the millisecond regime and a modest memory footprint [13,15,16]. In contrast, variants of R-CNN, while superior in localization precision [14,18], require a two-stage cascade of region proposal and per-region refinement. This results in computational overheads incompatible with edge-grade hardware [20,21]. Transformer-based detectors such as real-time detection transformer (RT-DETR) partially alleviate small-object omissions, yet their quadratic attention complexity and large activation maps remain prohibitive for resource-constrained deployments [12,17,22]. Given these limitations, a key research direction is to enhance the YOLO architecture’s feature extraction capabilities for multiple anomaly types while balancing detection accuracy and computational efficiency.

Currently, utility-scale PV plant inspections typically employ drones equipped with collinear RGB and thermal cameras to capture complementary spectral cues [23,24]. In practical applications, snow, stains, and defects are all significant causes of reduced power generation efficiency of PV panels, with defects potentially leading to thermal runaway and causing safety accidents such as fires. These faults often occur simultaneously in PV systems, and different types of faults require different treatment methods and resources. Nevertheless, as evidenced by the aforementioned research findings, existing algorithms focus solely on detecting specific defect types, lacking the capability for integrated detection of multiple types of faults. Therefore, we propose an innovative multi-anomaly detection framework, which integrates the YOLOv11 backbone network with the Mix Structure Block and the Outlook Attention mechanism, named YOLOv11-MO. This approach can process RGB and thermal images in parallel to simultaneously segment stains, internal defects, and snow occlusions, and specifically achieve coupled detection of bird droppings and the hotspots they induce.

The key innovations of this manuscript are summarized as follows:

(1) This research meticulously devises an advanced model, YOLOv11-MO, tailored explicitly for photovoltaic multi-fault detection. By incorporating the C2PSA-OutlookAttention (C2PSA-OA) module, it can accurately delineate the contours of micro-defects. Additionally, a mixed structure block (MSB) is embedded between the neck and head of the model, significantly enhancing the detection capability for small and indistinct defects. These optimizations not only markedly improve the model’s detection accuracy and robustness but also adeptly balance detection precision with computational efficiency.
(2) A bimodal RGB-thermal imaging dataset comprising 6,128 images captured by UAVs has been constructed, covering a variety of photovoltaic fault types, including stains, internal defects, and snow coverage. To augment data diversity and model generalizability, a suite of data enhancement strategies, including random rotation, translation, and contrast adjustment, has been applied to the images. Moreover, the HSV color space has been utilized to separate luminance from chrominance, thereby effectively enhancing the color features of fault regions and bolstering the model’s adaptability to complex lighting conditions and generalizability across diverse scenarios.
(3) YOLOv11-MO has been comprehensively compared with mainstream models, including YOLOv5, YOLOv8, YOLOv10, YOLOv12, and RT-DETR. The experimental results demonstrate that YOLOv11-MO outperforms other models in detection accuracy across all fault categories. Furthermore, the gradient-weighted class activation mapping (Grad-CAM) technique has been employed to visualize the model’s attention distribution, vividly illustrating its ability to precisely focus on fault-prone areas and thereby significantly enhancing the model’s interpretability.

The remainder of this manuscript is organized as follows: Section 2 provides a detailed exposition of the YOLOv11 detection architecture; Section 3 delves into the research context and offers a comprehensive description of the proposed model framework; Section 4 presents the experimental results and analyses, along with comparisons of detection accuracy and model size; and finally, Section 5 summarizes the manuscript and outlines future research directions.

## 2. YOLOv11 Detection Framework

**Figure 1:**
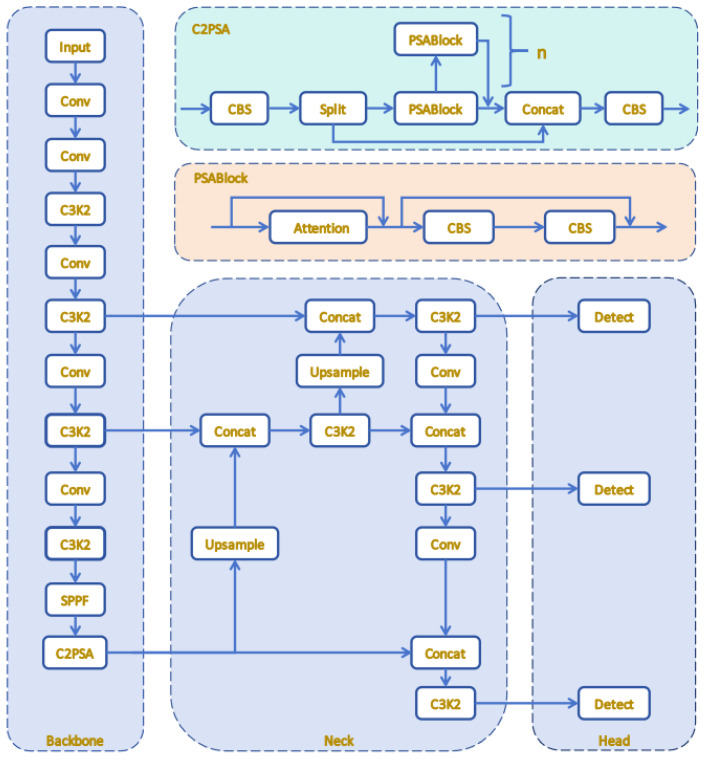
Holistic architecture of the YOLOv11 detection framework.

Target recognition methodologies have transitioned from traditional two-stage models to streamlined single-stage architectures, with the YOLO framework being a prime example. In the realm of PV fault detection, where swift real-time processing on edge devices is crucial and datasets are often limited, single-stage algorithms offer significant advantages due to their high computational efficiency and minimal risk of overfitting [25]. Considering these factors, YOLOv11, an advanced object detection framework, has been chosen as the core model for this research. The comparative results detailed in Section 4.3 illustrate that YOLOv11 demonstrated exceptional performance across the established PV fault detection dataset, surpassing other state-of-the-art algorithms, including YOLOv5 [26], YOLOv8 [27], YOLOv10 [28], YOLOv11 [25], YOLOv12 [29], and RT-DETR [22]. Moreover, YOLOv11 surpassed the latest YOLOv12 in this task. Its advantages lie in the improved dynamic anchor allocation and optimized gradient flow within the C3K2 module, which together enhanced the recall of small, low-contrast defects while maintaining low latency. Compared with previous versions, YOLOv11 continues to utilize its classic three-part architecture, namely the main network, connection module, and detection module. The main network employs C3K2 modules, significantly enhancing feature extraction efficiency through the Bottleneck Block, Spatial Pyramid Pooling Module (SPPF), and C2PSA modules. The connection module is responsible for feature integration and enhancement. The detection module, as the core decision-making unit of YOLOv11, is tasked with generating the final detection results. Figure 1 presents the holistic architecture of the YOLOv11 detection network. The architecture encompasses a main network for feature extraction, a connection module for feature fusion, and a detection module for object localization and classification. These modules operate in concert to ensure that the network processes input data efficiently and generates accurate detection results.

**Figure 2:**
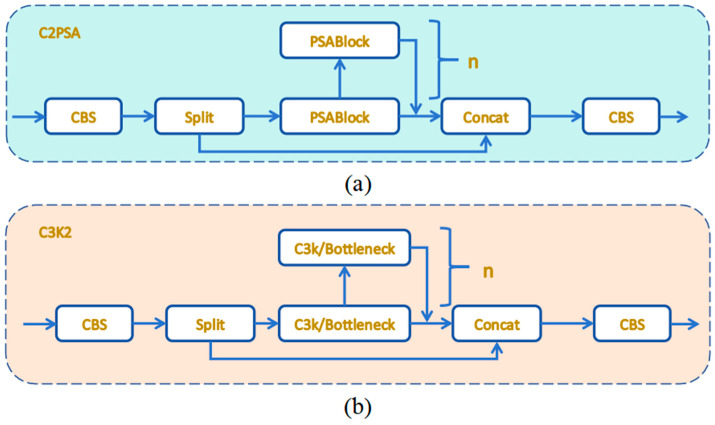
(**a**) C2PSA module. (**b**) C3K2 module.

YOLOv11 primarily optimizes the C3k2 and C2PSA modules in its model algorithm, significantly enhancing its feature extraction capabilities and improving the accuracy of multi-scale target recognition. The cross-scale pixel spatial attention (C2PSA) module in YOLOv11 incorporates a novel attention mechanism designed to refine feature representation by integrating cross-scale attention with pixel-level spatial optimization. As depicted in Figure 2a, the architecture of this module consists of an initial convolutional layer, a sequence of cascaded pixel spatial attention blocks (PSABlocks), a feature concatenation step (Concat), and a concluding convolutional layer dedicated to feature integration [30,31]. The initial convolutional layer is tasked with extracting fundamental features from the input data. Subsequently, these features are processed through a series of PSABlocks, each of which refines the feature representation iteratively. Each PSABlock features a pixel spatial attention mechanism paired with a residual connection. This attention mechanism employs a weighting strategy to highlight critical features within target regions, thereby diminishing background noise and enhancing the model’s focus on salient details. Additionally, the residual connections ensure smooth gradient flow. The C2PSA module’s integration of cross-scale attention and pixel-level optimization effectively balances the preservation of fine details with the broader context, rendering it highly effective for detecting complex objects in challenging environments.

The C3K2 module functions as a crucial feature extraction unit in YOLOv11, adeptly balancing feature representation and computational efficiency via a multibranch architecture and residual connections. As depicted in Figure 2b, the C3K2 module provides two distinct configurations. When the parameter c3k is set to False, the module utilizes a standard bottleneck structure, enhanced by supplementary convolutional and activation layers to extract deeper and more complex features. Conversely, when c3k is set to True, the bottleneck structure is replaced with a C3 module, which significantly reduces computational complexity [32,33]. The multibranch architecture of the C3K2 module enables efficient extraction of multi-scale features, enhancing its adaptability to contemporary object detection tasks and ensuring robust performance across various scenarios.

## 3. Methods

**Figure 3:**
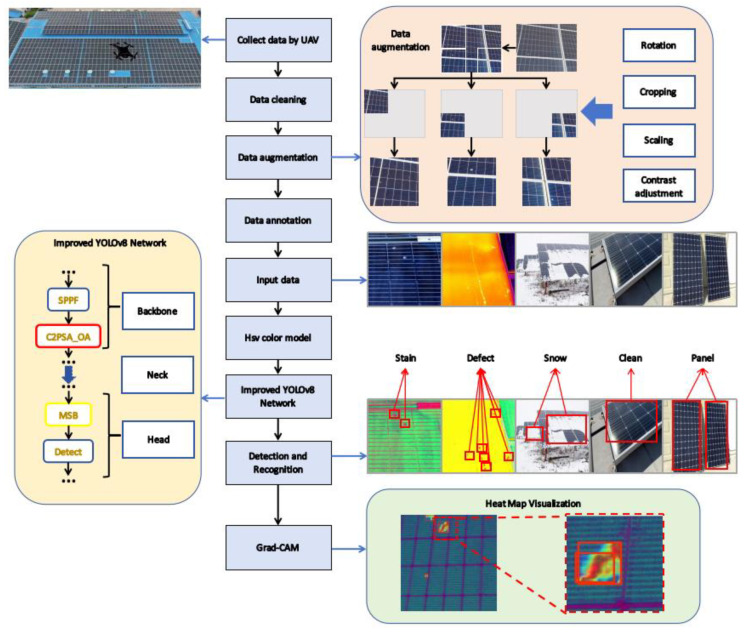
Schematic diagram of the proposed methodology.

Utilizing a UAV platform, we captured co-registered RGB and thermal imagery of rural photovoltaic installations and constructed a comprehensive five-category fault dataset encompassing stains, defects, snow cover, and two auxiliary labels, along with a specialized subset tailored for the concurrent detection of bird-dropping contamination and hotspot anomalies. By mapping images into the HSV color space, we enhanced the color discrepancies within fault regions, thereby improving the discriminability of features. Subsequently, we designed a detector that integrates MSB and Outlook Attention (OA) modules. Although this led to an increase in model parameters, it provided more accurate multi-class localization and enhanced feature extraction capabilities. Figure 3 illustrates the entire research workflow.

### 3.1. Data Collection

Field campaigns were conducted at two sites: (i) rural PV installations in Yushan District, Ma’anshan, Anhui (Longitude 118.49, Latitude 31.68) and (ii) utility-scale plants near Zhengzhou, Henan (Longitude 113.39, Latitude 34.43). The DJI Mavic 3T thermal imaging drone employed in this study maintains stable performance under extreme high-temperature conditions and features high-resolution wide-angle and infrared cameras for acquiring RGB and infrared images. Moreover, its compact design minimizes light obstruction during image capture [34,35,36]. To preserve radiometric fidelity, flights were scheduled around 15:00 under clear skies; multi-angle and variable-illumination shots were acquired for diversity. The complete dataset comprises 1468 images, including 120 infrared images and 1348 RGB images, archived in JPEG format. During the data collection process, since no snow-covered weather was encountered in the field, the data related to snow coverage were sourced from publicly available online datasets. Key flight parameters are listed in Table 1.

### 3.2. Data Curation and Labeling

After filtering the photovoltaic images captured by the UAV, we obtained 1292 RGB images and 109 infrared images. Despite efforts to minimize the impact of illumination variations, shooting angle deviations, and environmental disturbances during data collection, these factors can still interfere with the accuracy of subsequent detection. To address these issues, the following preprocessing steps were performed in sequence: data cleaning, contrast adjustment, cropping and scaling, and data annotation [37,38,39].

**Figure 4:**
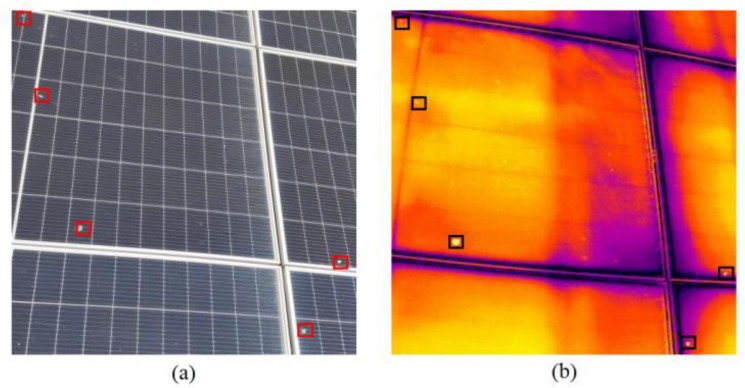
(**a**) RGB image. (**b**) Infrared image.

To ensure data quality, we initially performed data preprocessing, eliminating out-of-focus and repetitive images, thereby significantly augmenting the precision and dependability of model training and forecasting. Subsequently, we applied stochastic rotation and displacement to the imagery to emulate a variety of viewing perspectives and contexts, effectively enhancing the algorithm’s adaptability. Additionally, we adjusted visual attributes like luminance, contrast, and chroma to simulate different lighting conditions. Considering the resolution differences between infrared and RGB imagery, we conducted random cropping and scaling, using the image center as a reference to resize all visual data to a dimension of 640 × 640 pixels. After these processes, we used Roboflow [40] to precisely annotate the visual data, furnishing high-quality educational data for ensuing identification and dissection. Figure 4 illustrates some exemplary RGB and infrared imagery.

As shown in Figure 4, the bird-dropping contamination in Figure 4a and the hotspot defects in Figure 4b are spatially correlated. The RGB image can accurately locate the bird-dropping contamination areas, while the infrared image can simultaneously capture the hotspot locations caused by the shading of bird droppings. Therefore, the joint detection of these two faults can provide a basis for precise fault handling and effectively enhance the operational efficiency and reliability of photovoltaic modules.

### 3.3. HSV Color Model

In the context of multi-fault detection in photovoltaic modules, the model is required to simultaneously identify bird-dropping contamination and hotspot defects, both of which are spatially correlated and significantly impact the performance of PV modules. However, when attempting to integrate RGB images and infrared images for comprehensive detection, challenges arise due to the differences in sensor data. Specifically, RGB images primarily reflect color information, while infrared images mainly reflect temperature information. The significant differences in physical meaning and data characteristics between the two types of images make direct integration difficult. To overcome this challenge and effectively integrate the information from both types of images, this study introduces the HSV color model [41,42]. This model refines color information into three key dimensions: hue, saturation, and value, thereby effectively separating color and illumination information while highlighting temperature differences. Through this decomposition, the model can more accurately extract color features related to bird-dropping contamination and enhance the recognition of temperature changes in hotspot areas, providing a more accurate and reliable basis for fault detection in PV modules. The Equations (1)–(3) are the transformation formulas of the HSV color model:

(1) $$H=\left\{\begin{array}{c} 60\times \frac{G-B}{\left(R-G\right)+\left(R-B\right)},R=Max(R,G,B)\\ 60\times \frac{B-R}{\left(G-R\right)+\left(G-B\right)},G=Max(R,G,B)\\ 60\times \frac{R-G}{\left(B-G\right)+\left(B-R\right)},B=Max(R,G,B) \end{array}\right.$$

(2) $$S=\frac{Max(R,G,B)-Min(R,G,B)}{Max(R,G,B)}$$

(3) V=Max(R,G,B)

**Figure 5:**
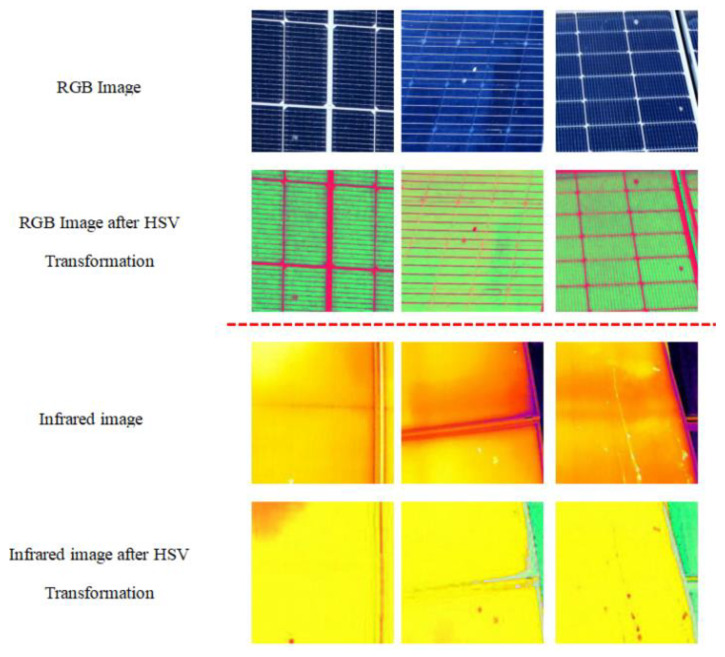
Comparative analysis of RGB and imagery following HSV color space transformation.

The RGB color space is an additive color model based on light, generating various colors by combining red (R), green (G), and blue (B) light at different intensities. However, it is highly sensitive to lighting conditions, with color perception significantly influenced by ambient illumination. Each component of R, G, and B has a value range of [0, 255]. After conversion to the HSV color space, colors are represented by (H,S,V), where H, S, and V denote hue, saturation, and value, respectively. Hue and saturation are more suitable for color feature extraction, while value is better for extracting lighting features. Figure 5 visually illustrates the visual changes in stains and hotspots on photovoltaic module images during the HSV transformation process.

Figure 5 visually demonstrates the effect of the HSV color space transformation. This transformation enhances the color features associated with bird-dropping contamination by separating the hue and saturation channels, thereby improving recognition efficiency. In the case of infrared images, thermal anomalies are clearly highlighted through the value channel, significantly enhancing the visibility of hotspot defects [43]. Overall, this color space transformation significantly enhances the model’s adaptability and stability across different sensors and lighting conditions. More importantly, it facilitates the extraction of key diagnostic features specific to photovoltaic modules, enabling more accurate and reliable fault detection without missing critical patterns under complex and noisy conditions.

### 3.4. Enhanced YOLOv11 Framework

**Figure 6:**
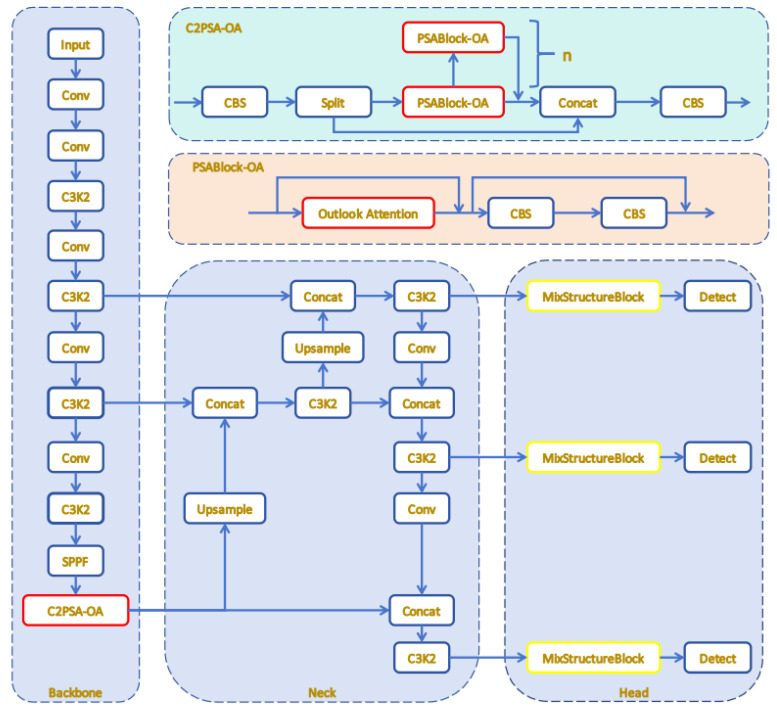
YOLOv11-MO architecture.

In the realm of PV fault detection, YOLOv11 exhibits limitations, such as weak fault adaptability, poor generalization capability, and inadequate feature representation. To tackle these limitations, the current research presents an improved YOLOv11-MO algorithm, which integrates the OA [44] and MSB [45] modules. As depicted in Figure 6, the architecture of the YOLOv11-MO algorithm is illustrated.

OA dynamically aggregates local features via a sliding window, replacing the original Attention in C2PSA, and effectively captures fine-grained details often overlooked by traditional self-attention mechanisms, thereby enhancing semantic understanding. The MSB module fuses features across different scales using multi-scale parallel large convolution kernels and combines various convolution operations to improve feature discriminability. This is crucial for distinguishing between fault types with distinct spatial and textural characteristics. Additionally, the module incorporates parallel attention mechanisms that focus on different aspects of the input features simultaneously, effectively handling uneven fault distributions and ensuring that both global and local features are adequately represented. These improvements enable YOLOv11-MO to be capable of significantly enhancing detection accuracy and generalization ability while maintaining the real-time inference capability of YOLOv11, thereby enabling more precise identification of various types of faults. Additionally, the original activation function of YOLOv11 is replaced with the bounded sigmoid linear unit (B-SiLU) [46]. This substitution introduces complex feature interactions, enabling more effective capture and representation of key features in the input data, thereby enhancing detection accuracy.

#### 3.4.1. Outlook Attention

In UAV high-altitude inspection scenarios, PV modules exhibit three challenging characteristics—extreme scale variation, irregular geometry, and random occlusion. In the original YOLOv11 model, the native multi-head self-attention in C2PSA is redundant and overly global, causing the model to focus on background regions and consequently lose sensitivity to tiny defects, particularly when millimeter-sized bird droppings coexist with meter-scale snow coverage, and micro-cracks are easily submerged in texture noise. To address this issue, we integrate OA of Vision Outlooker (VOLO), which performs linear-complexity self-attention within 3 × 3 sliding windows [44]. The core computation is

(4) $$OutlookAttn\left(X\right)=Fold\left(Softmax\left(\frac{{QK}^{T}}{\sqrt{d}}\right)\right)+X$$

where the linear-window design significantly reduces memory footprint and computational load while enhancing fine-grained representation of edges and textures, enabling accurate multi-scale defect detection under stringent edge-device budgets.

**Figure 7:**
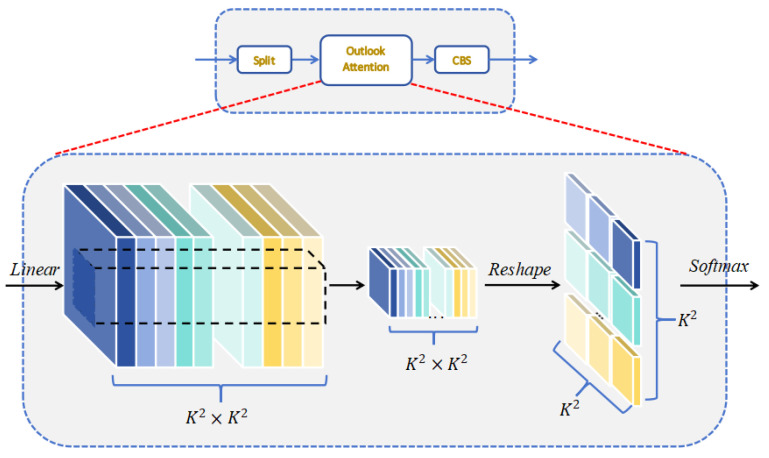
Architecture of the Outlook Attention.

The detailed workflow of OA is illustrated in Figure 7. An input feature map $X\in R^{H\times W\times C}$ is first unfolded via a 3 × 3 window with stride 1 into local patches of shape (H × W) × 9 × C. As shown in Equation (5), a shared linear projection $W_{q,k,v}\in R^{C\times C}$ generates Query, Key, and Value tensors. Attention weights are then calculated and applied to each window. The resulting features are folded back to the original spatial resolution and added to the residual path, followed by a two-layer multi-layer perceptron (MLP) for channel-wise refinement. Owing to local matrix multiplication, overall complexity grows linearly with window size, yielding an order-of-magnitude reduction in memory consumption compared with global self-attention.

(5) $$Q,K,V=Unfold\left(X\right)W_{q,k,v}$$

#### 3.4.2. Mix Structure Block

Image quality also plays an important role in PV fault detection. High-altitude images taken by UAV often suffer from quality degradation due to haze, snow, and uneven lighting, resulting in blurred or missing local details. The original YOLOv11 backbone is constrained by its single-scale receptive field and serial attention, making it difficult to simultaneously capture large-area defects and fine-grained faults.

MSB integrates the lightweight Multi-Scale Parallel Large Convolution Kernel and Enhanced Parallel Attention sub-modules [45]. The Multi-Scale Parallel Large Convolution Kernel employs three depth-wise dilated convolutions with kernels 19, 13, and 7 and dilation 3 in parallel, concatenates their outputs and passes the result through a GELU-activated MLP to restore both global haze regions and fine textures. Enhanced Parallel Attention runs simple pixel, channel and pixel attentions concurrently to encode global context and location-specific details; the three attention maps are concatenated, dimension-reduced by an MLP and added to the residual.

**Figure 8:**
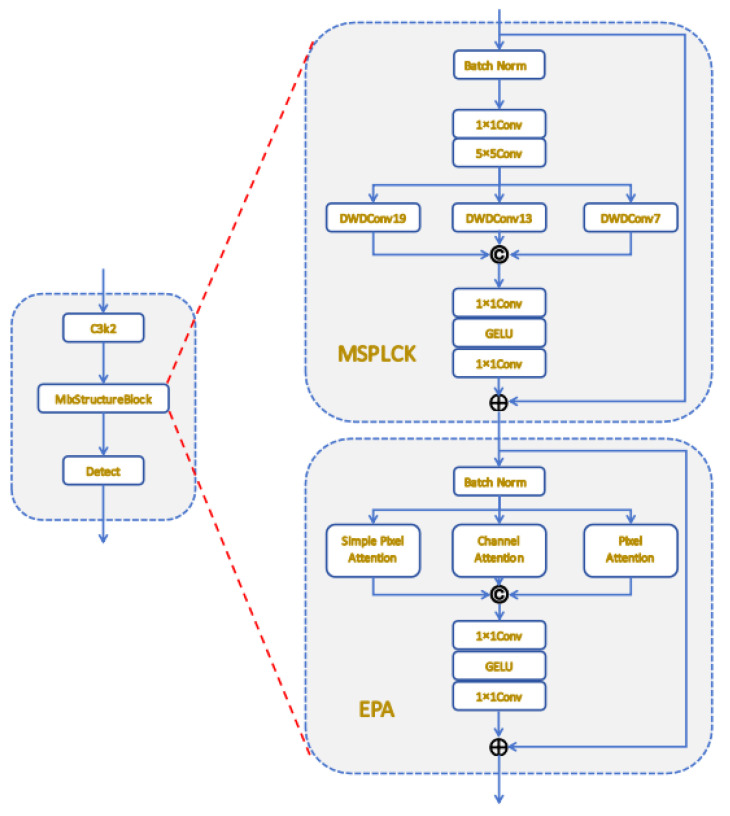
Design of the Mix Structure Block module.

The overall MSB computation is

(6) $$F_{out}=x+MLP\left(Concat\left[MSPLCK\left(x\right),\;EPA\left[x\right]\right]\right)$$

where *x* denotes the input feature map. After embedding the MSB, the model’s detection accuracy is improved, enabling robust identification of snow, stains, and defects under complex aerial conditions. Consequently, the MSB is strategically positioned between the neck and head of YOLOv11, thereby augmenting the model’s capacity to discern features across multiple scales, with the detailed structure depicted in Figure 8.

#### 3.4.3. B-SiLU

Activation functions serve as a crucial element in neural networks, imparting nonlinearity that empowers the model to capture intricate feature representations. Within the realm of deep learning, the selection of activation functions markedly influences the model’s efficacy, training efficiency, and rate of convergence. In pursuit of augmenting the detection accuracy and robustness of the YOLOv11 model within intricate scenarios, this study introduces a novel activation function—B-SiLU [46]. B-SiLU integrates the self-gating characteristics of SiLU and the smooth gradient properties of GELU, while further enhancing the model’s training process and performance through the introduction of boundedness. Its mathematical expression is as follows:

(7) $$B-SiLU\left(x\right)=\left(x+\alpha \right)\cdot \sigma \left(x\right)-\frac{\alpha }{2}$$

Here, $\sigma \left(x\right)$ denotes the Sigmoid function, which is defined as

(8) $$\sigma \left(x\right)=\frac{1}{1+e^{-x}}$$

The parameter α is a tunable parameter used to control the lower bound of the activation function. By introducing α, B-SiLU can maintain non-zero gradients for negative inputs, thereby effectively avoiding the vanishing gradient problem. Meanwhile, the smooth gradient characteristics of B-SiLU help improve the optimization process, enhancing the model’s convergence speed and stability. In the task of photovoltaic multi-fault detection, the model needs to accurately identify and locate various types of faults. These fault types have different spatial and textural features and often appear under complex backgrounds and lighting conditions. Therefore, the robustness and accuracy of the model are crucial. By introducing B-SiLU, the network ensures non-zero gradients for negative inputs, enabling the network to more effectively utilize negative input values and improve the model’s robustness against complex backgrounds and noise. Meanwhile, the smooth gradient characteristics of B-SiLU help reduce the risk of model overfitting, significantly enhancing the model’s robustness and reliability in practical applications.

### 3.5. Grad-CAM-Based Model Decision Visualization

We employed the Grad-CAM technology to interpret the effectiveness of neural network models in detecting multiple types of photovoltaic module faults [47,48]. This technique utilizes the gradient information flowing into the final convolutional layer to generate visual explanations. It retains detailed spatial information while capturing high-level semantic features, thereby offering valuable insights into the model’s decision-making rationale.

Initially, the input image is passed through the model via forward propagation to obtain the feature maps of the target layer. These feature maps serve as intermediate representations of the input image within the model, encapsulating the model’s interpretation of the image. Subsequently, backpropagation is conducted from the predicted score of the target class to acquire the gradients of the feature maps in the target layer. These gradients reflect the contribution of each element in the feature maps to the specific type of fault. To further quantify the significance of each feature map, global average pooling is applied to the gradients of each feature map, yielding the weights for each feature map:

(9) $$\alpha_{k}^{c}=\frac{1}{H\times W}\sum_{i=1}^{H}\sum_{j=1}^{W}\frac{\partial y_{c}}{\partial A_{ij}^{k}}$$

Here, $\alpha_{k}^{c}$ represents the weight of the k-th feature map for class c, and $\frac{\partial y_{c}}{\partial A_{ij}^{k}}$ denotes the gradient of the predicted score for class c with respect to the feature map $A_{ij}^{k}$. This gradient reflects the importance of each element in the feature map to class c. Finally, each feature map is multiplied by its corresponding weight, and the results are summed and then passed through the ReLU function to obtain the class activation map:

(10) $$L_{Grad-CAM}^{c}=ReLU\left(\sum_{k=1}^{C}\alpha_{k}^{c}A^{k}\right)$$

The resultant heatmap intuitively highlights the regions that the model focuses on when detecting specific types of faults, thereby not only enhancing the interpretability of the model but also providing strong support for its optimization and practical application.

## 4. Results and Discussion

### 4.1. Dataset Acquisition and Experimentation

#### 4.1.1. Dataset Arrangement

**Figure 9:**
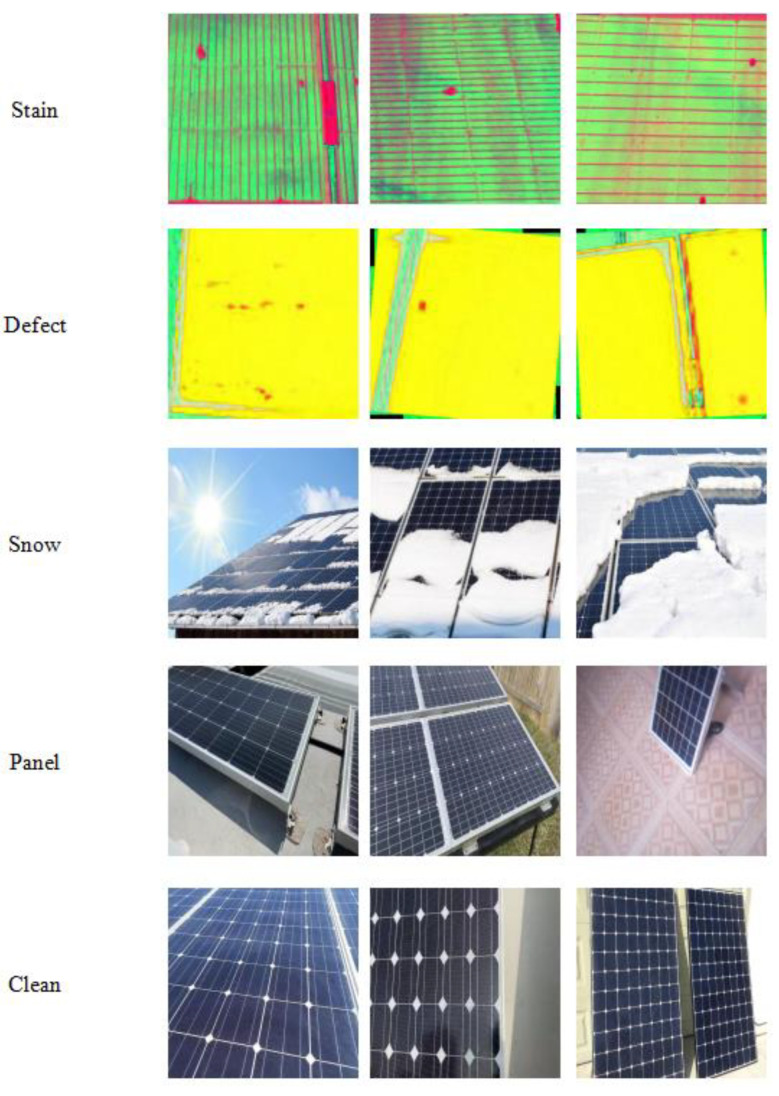
Representative images of the five categories within the dataset.

Following the data acquisition, enhancement, and annotation procedures detailed in Section 3, a multimodal dataset comprising 6128 images was constructed, partitioned into training (70%), validation (10%), and testing (20%) subsets. This dataset encompasses a variety of common photovoltaic anomalies, including stains, internal defects, snow coverage, panel conditions, and clean states, rendering it suitable for diverse fault detection tasks, with detailed information presented in Table 2. Additionally, the dataset includes a smaller merged subset with 1289 hotspot labels and 5963 bird-dropping labels. Figure 9 illustrates representative images of the five categories within the dataset.

**Figure 10:**
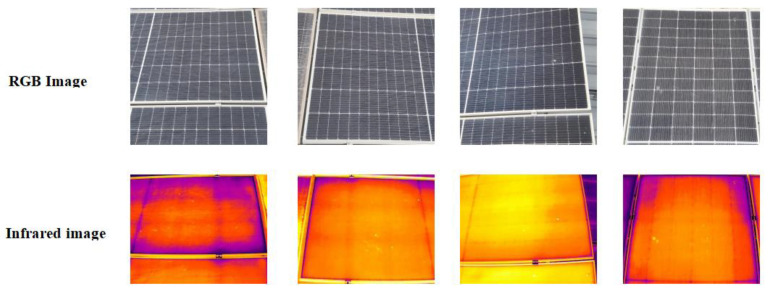
A selection of raw images captured by the drone.

In this study, the small-scale fusion dataset includes RGB images and corresponding infrared images captured by an infrared dual-spectrum drone. Additionally, extra RGB images containing only bird-dropping features (without infrared counterparts) were collected to enhance the model’s ability to recognize bird-dropping colors and shapes. The dataset creation process is as follows. First, using the image center as a reference, RGB images (4000 × 3000 pixels) and infrared images (640 × 512 pixels) are cropped and resized to the same dimensions (640 × 512 pixels) to ensure spatial alignment. Then, bird-dropping and hot-spot regions are annotated. After that, the annotated images are processed with the HSV color model to enhance bird-dropping color features and extract hot-spot temperature features. Finally, the processed RGB and infrared images are integrated into a single dataset. For RGB images with corresponding infrared images, infrared temperature features are introduced. For extra RGB images without infrared counterparts, they are annotated as bird-dropping regions to increase the model’s learning samples of bird-dropping features. Figure 10 shows the raw data captured by the drone.

#### 4.1.2. Experimental Configuration

In this work, each test was conducted using the same hardware and software setup. Specifically, the experiments were performed on a single RTX A5000 GPU with a software environment comprising CUDA 12.5, Python 3.10, and PyTorch 2.4.0. Based on preliminary experiments, the SGD optimizer with an initial learning rate of 0.01 was employed [49,50]. During the training phase, the input images were resized to 640 × 640. The batch size for model training was set to 8. All experiments were trained for 150 epochs to ensure robust convergence across all models.

### 4.2. Evaluation Metrics

To comprehensively evaluate the detection performance of the YOLOv11-MO model for multiple types of photovoltaic panel faults, a suite of evaluation metrics was employed, including precision, recall, mean average precision (mAP), frames per second (FPS), and the total parameter count of the model [51]. Among these evaluation metrics, true positives (TP) refer to the correctly predicted positive samples, false positives (FP) refer to the incorrectly predicted positive samples, and true negatives (TN) refer to the correctly predicted negative samples.

Precision is the ratio of TP to the sum of TP and FP, as shown in Equation (11), where TP represents the number of correctly identified positive samples, and (TP + FP) represents all predicted positive samples.

(11) $$Precision=\frac{TP}{TP+FP}$$

Recall is the ratio of TP to the sum of TP and FN, as shown in Equation (12), where TP represents the number of correctly identified positive samples, and (TP + FN) represents all actual positive samples.

(12) $$Recall=\frac{TP}{TP+FN}$$

Average precision (AP) amalgamates precision and recall across diverse thresholds, signifying the model’s aptitude in recognizing objects within a specific category. For multi-category datasets, the mean average precision (mAP) is derived by averaging the AP scores across all classes:

(13) $$AP=\int_{0}^{1}P\left(R\right)dR$$

(14) $$mAP=\frac{1}{C}\sum_{t=1}^{C}AP_{t}$$

FPS denotes the quantity of images that can be processed within a unit of time. Given identical hardware resources, a higher FPS of the object detection algorithm correlates with superior real-time performance.

The forthcoming section details the experimental results, utilizing the aforementioned metrics and parameters.

### 4.3. Comparative Experiments

In order to meticulously evaluate the effectiveness of the proposed algorithm, comparative experiments were conducted against several state-of-the-art object detection models, employing identical datasets and training protocols. Considering that inspections of photovoltaic panels are often carried out using UAVs, which demand compact models and efficient computational performance, lightweight versions of the YOLO series were selected for benchmarking. The outcomes of the aforementioned experiments are encapsulated in Table 3 and Table 4.

As shown in Table 3, YOLOv11 demonstrates significant superiority in key performance metrics, achieving an mAP@0.5 of 0.798. These metrics not only represent the best performance within the YOLO series but also markedly outperform RT-DETR. The outstanding performance of YOLOv11 is primarily attributed to the introduction of the C2PSA and C3K2 modules. The C2PSA module, through its cross-stage partial attention mechanism, significantly enhances the model’s ability to detect small objects and complex backgrounds. Meanwhile, the C3K2 module further optimizes the feature extraction process, thereby improving the overall performance of the model. In contrast, YOLOv5, YOLOv8, YOLOv10, and YOLOv12 all exhibit lower performance than YOLOv11, indicating that these models still have room for improvement in feature extraction and target detection. Although YOLOv12 shows relatively strong robustness overall, it still lags behind YOLOv11 in certain categories, suggesting that its architecture and feature extraction capabilities warrant further refinement. RT-DETR’s performance is mediocre, with an mAP@0.5 of 0.702. Despite the advantage of RT-DETR’s Transformer structure in handling long-range dependencies, it performs poorly in detecting small objects and processing complex backgrounds, resulting in lower detection accuracy and robustness compared to YOLOv11.

A more in-depth analysis of performance across different categories is provided in Table 4. YOLOv11 outperforms other models in all categories, particularly in the Clean and Defect categories, where its mAP@0.5 reaches 0.965 and 0.813, respectively. This indicates that YOLOv11 has extremely high accuracy and robustness in detecting clean and defective photovoltaic panels. For the Stain category, mAP@0.5 of YOLOv11 is 0.615; while this represents competent performance, it remains notably lower than other categories. This gap is likely attributable to the complex types and distributions of dirt in the Stain category, which require further optimization of the feature extraction module to enhance detection performance. In the Panel and Snow categories, the mAP@0.5 of YOLOv11 are 0.766 and 0.833, respectively, both significantly higher than other models. These results demonstrate YOLOv11’s high accuracy in detecting the overall photovoltaic panel and snow coverage. further confirming its comprehensive advantages in handling different types of PV faults.

In summary, YOLOv11 performs exceptionally well in photovoltaic fault detection tasks, especially in detecting Clean, Defect, and Snow categories, with high accuracy and robustness. However, there is still room for improvement in the Stain category. Therefore, YOLOv11 was selected as the model for improvement, with the aim of optimizing the model structure and enhancing feature extraction capabilities to further improve detection performance for complex backgrounds and small objects.

### 4.4. Ablation Experiment

In order to systematically assess the contributions of the individual enhancements proposed for YOLOv11, a series of incremental assessments were conducted. Initially, the efficacy of incorporating the HSV color model was examined. This was followed by ablation studies on the MSB and OA modules, which were integrated into YOLOv11 in conjunction with the HSV model. Finally, the impact of introducing the B-SiLU activation function was assessed. The cumulative results of these evaluations are compiled in Table 5 and Table 6.

Table 5 provides detailed results of the ablation study, revealing the significant impact of different module combinations on model performance. When the HSV module is introduced alone, the mAP@0.5 slightly increases to 0.799, while the recall rate slightly decreases to 0.762, and the precision drops to 0.821. The model size remains at 18.3 MB, and the FPS increases from 17.441 to 18.398. This indicates that the HSV module has certain advantages in enhancing color feature extraction, but it has a slight negative impact on recall rate. Meanwhile, the HSV module has a minimal impact on computational resources, with an increase in FPS. After further introducing the Outlook Attention module, the mAP@0.5 increases to 0.801, the recall rate slightly decreases to 0.766, and the precision drops to 0.814. The model size remains at 18.3 MB, and the FPS increases from 17.441 to 18.320. This suggests that the Outlook Attention module significantly enhances the model’s detection capability for small targets and complex backgrounds. When the Hybrid Structure Block module is introduced, the mAP@0.5 increases to 0.804, the recall rate increases to 0.770, and the precision slightly drops to 0.817. The model size increases to 59.3 MB, and the FPS decreases from 17.441 to 14.282. This highlights the significant advantages of the Hybrid Structure Block module in handling complex backgrounds and small targets, although the computational complexity increases. Ultimately, when all modules (HSV, Outlook Attention, Hybrid Structure Block, and B-SiLU) are integrated, the mAP@0.5 reaches 0.816, with a recall rate of 0.773 and precision of 0.835. The model size remains at 59.3 MB, and the FPS is 14.637. This emphasizes the significant overall performance improvement brought by the synergistic effect of these modules, especially in the Defect, Stain, Panel and Snow categories.

As shown in Table 6, the impact of different modules on performance varies across categories. In the Clean category, the base model’s mAP@0.5 is 0.965, which slightly decreases after the introduction of the HSV module but gradually increases to 0.961 and finally reaches 0.949 with all modules incorporated. This indicates that the Clean category has a relatively low dependence on additional modules, as the base model already achieves high detection accuracy. In contrast, the Defect category is more sensitive to module introduction. The base model’s mAP@0.5 is 0.813, which drops to 0.779 upon the addition of the HSV module but subsequently rises to 0.835. Notably, the introduction of the Outlook Attention and Mix Structure Block modules notably improves detection performance. The Stain category also shows sensitivity to module introduction. The baseline model achieves an mAP@0.5 of 0.615, which fluctuates between 0.615 and 0.651 with different modules and ultimately reaches 0.648 with all modules included. Similarly, the Panel category exhibits sensitivity to module introduction. The baseline model achieves an mAP@0.5 of 0.766, which varies between 0.757 and 0.811 with different modules and finally reaches 0.808 with all modules incorporated. The Snow category is likewise sensitive to module introduction. The baseline model achieves an mAP@0.5 of 0.833, which fluctuates between 0.828 and 0.853 with different modules and ultimately reaches 0.841 with all modules included. These findings indicate that different categories have varying sensitivities to module introduction, with specific modules significantly enhancing detection performance in the Defect, Stain, Panel, and Snow categories.

From the results in the above tables, one may infer that the integration of various modules markedly augments the overall performance of the model, particularly in addressing complex backgrounds and small objects. The synergistic effect of these modules plays a crucial role in performance improvement, particularly in the Defect, Stain, Panel, and Snow categories. However, this performance gain comes at a cost. The introduction of the MSB module, while significantly boosting detection performance, also substantially increases the model’s computational complexity (model size increases from 18.3 MB to 59.3 MB). This suggests that a trade-off between performance enhancement and computational resource consumption needs to be considered in practical applications.

To effectively address the increased computational complexity, this study has adopted a series of strategies. First, the HSV color model was introduced to enhance the model’s capability in extracting color features. Second, the OA module was incorporated, which effectively captures fine-grained details often overlooked by traditional self-attention mechanisms. Lastly, the original activation function of YOLOv11 was replaced with the B-SiLU, introducing complex feature interactions that enable the model to more effectively capture and represent key features in the input data. As shown in Table 5, these improvements have all led to an increase in the model’s FPS, thereby alleviating the increase in computational complexity to some extent while maintaining high performance. Overall, despite the increase in model size, the decrease in FPS is relatively small, from 17.441 to 14.637, indicating that the impact on real-time performance is within an acceptable range.

Moreover, the varying sensitivity of different categories to module introduction indicates that the selection of module combinations should be tailored to specific task requirements in practical applications. Future research could further explore more efficient module designs to enhance performance without significantly increasing computational complexity. Additionally, employing a broader range of data enhancement methods could bolster the model’s resilience to diverse environmental contexts, thereby significantly augmenting its generalization capacity. Specifically, we plan to introduce advanced multi-modal data augmentation techniques, incorporating spectral and spatiotemporal information, to further optimize the model’s training process. We will also explore hybrid architectures that combine the strengths of different models to achieve more efficient and accurate detection.

### 4.5. Cross-Technology Evaluation

In modern PV systems, in addition to traditional monocrystalline and polycrystalline silicon PV modules, thin-film and bifacial PV modules are increasingly being utilized. These novel PV modules exhibit significant differences from traditional modules in terms of material properties, optical response, and thermal characteristics. Therefore, to validate the detection performance of the model on these new types of modules, the YOLOv11-MO model was trained and evaluated on the novel PV module fault dataset from Roboflow. The mAP, recall, and precision of the model were calculated for different types of PV modules. The results are shown in Table 7.

The dataset size varies significantly across different types of PV modules. Monocrystalline silicon modules, which hold a dominant market position and are widely installed, possess the largest dataset, comprising 6128 images. In contrast, thin-film and bifacial modules, which have smaller market shares and are less frequently installed and used, respectively, have smaller datasets, with 203 and 182 images each. These smaller datasets highlight the need for further expansion in the future.

In the evaluation of detection performance across different types of PV modules, we focused on three key metrics: mAP@0.5, recall, and precision. The results indicate that polycrystalline silicon modules achieved the highest mAP@0.5 of 0.867, demonstrating superior detection accuracy. Monocrystalline silicon modules followed with an mAP@0.5 of 0.816, which, although slightly lower than that of polycrystalline silicon, still represents a high level of performance. In contrast, thin-film and bifacial modules had lower mAP@0.5 values of 0.723 and 0.737, respectively. These lower values may be attributed to the uniform surface texture and low color contrast of thin-film modules, as well as the increased detection complexity due to the reflective properties of the rear side of bifacial modules. Regarding recall, polycrystalline silicon modules had the highest rate at 0.838, indicating that the model was able to detect a greater proportion of fault regions with fewer missed detections. Monocrystalline silicon modules had a recall of 0.773, which, while slightly lower, still represents a high detection capability. Thin-film modules had the lowest recall at 0.721, likely due to the challenging identification of fault regions on their surfaces. Bifacial modules had a recall of 0.833, comparable to polycrystalline silicon modules, suggesting good detection performance despite the added complexity of their reflective properties. In terms of precision, polycrystalline silicon modules again had the highest rate at 0.836, indicating the lowest false-positive rate among the fault regions detected. Monocrystalline silicon modules had a precision of 0.835, slightly lower but still high. Thin-film modules had the lowest precision at 0.671, with bifacial modules at 0.664. The lower precision values for thin-film and bifacial modules may be due to the increased detection complexity resulting from their surface characteristics, which can lead to higher false-positive rates.

Overall, the model exhibits notable performance disparities across different types of PV modules. Polycrystalline silicon modules achieve the highest performance in terms of mAP@0.5, recall, and precision, which may be attributed to their stable optical and thermal properties. In contrast, thin-film and bifacial modules demonstrate lower performance metrics, likely due to their surface characteristics and increased detection complexity. The small dataset size of thin-film modules may particularly limit the model’s training efficacy and generalizability. Although bifacial modules show relatively high recall, their precision is lower, possibly because the reflective properties of their rear side complicate detection. Future work should focus on expanding the dataset sizes for thin-film and bifacial modules to gain a more comprehensive understanding of the model’s performance.

### 4.6. Visual Interpretation via Grad-CAM Analysis

**Figure 11:**
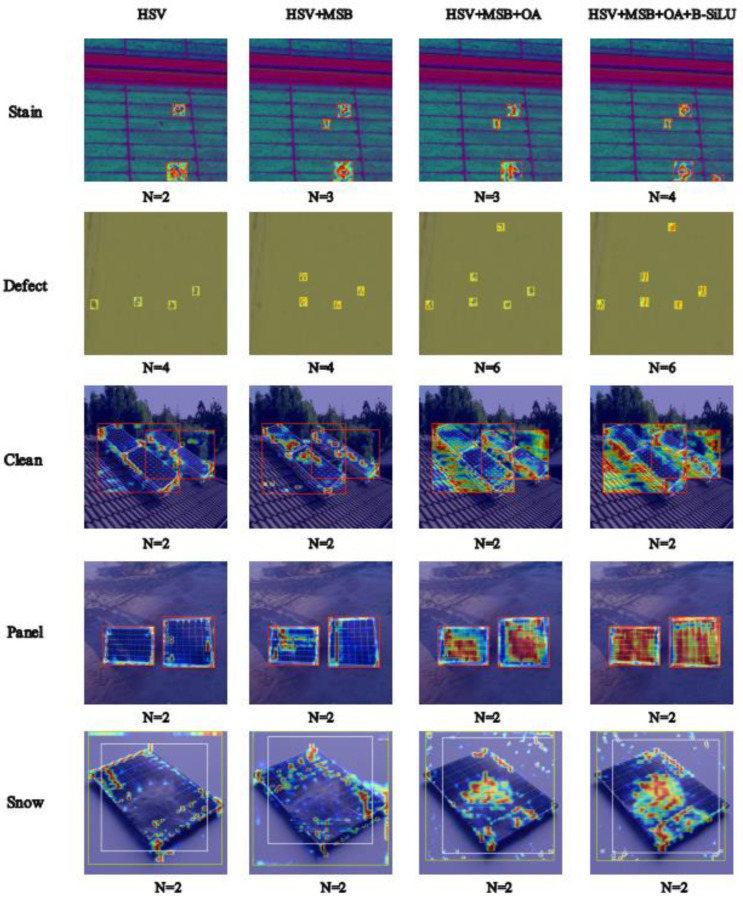
Grad-CAM analysis of attention heatmaps for YOLOv11s in photovoltaic fault detection.

In this study, we utilized the enhanced YOLOv11 model to generate heatmaps corresponding to different combinations of improvements, thereby visualizing the feature responses and attention distribution during the photovoltaic fault detection process. Figure 11 presents the heatmap outputs for five categories of photovoltaic faults (Dirty, Damaged, Clean, Panel, Snow), each under four distinct processing conditions: HSV, HSV + MSB, HSV + MSB + OA, and HSV + MSB + OA + B-SiLU.

The HSV module, which converts images from the RGB space to the HSV space, enhances sensitivity to color and brightness. In Figure 11, the heatmaps after HSV processing already demonstrate preliminary attention to target regions, particularly for the Stain and Defect categories, where the model can initially identify fault regions. However, relying solely on the HSV module still has limitations in detection capability, resulting in missed and false detections. To address this, the MSB module introduces a hybrid structural design that integrates feature extraction capabilities across different scales. In the heatmaps after HSV + MSB processing, the number of detection boxes for the Stain category increased from 2 to 3. This improvement indicates that the MSB module, through its synergistic design of multi-dilation-rate convolutions and channel shuffling, significantly enhances the model’s ability to capture cross-scale features and effectively suppresses missed detections caused by target scale differences. Building on these enhancements, the OA module further boosts the attention mechanism. In the heatmaps after HSV+MSB+OA processing, the model exhibits heightened attention to target regions of the Clean, Panel, and Snow categories, with more concentrated color distribution. The OA module achieves a balance between computational efficiency and long-range dependency modeling by integrating fine-grained attention within local windows and neighboring information, thereby enhancing the model’s perception of targets in complex backgrounds. Finally, the B-SiLU activation function, which combines the advantages of ReLU and SiLU, introduces nonlinear characteristics that enhance the model’s feature expression capability. In the heatmaps after HSV + MSB + OA + B-SiLU processing, the detection performance of the model reaches its optimal state, with the number of detection boxes for the Stain category increasing from 3 to 4. This improvement underscores the role of the B-SilU activation function in optimizing gradient flow and feature discrimination.

In conclusion, each improvement module has played a crucial role in enhancing the detection performance of the model. The HSV module provides basic sensitivity to color and brightness; the MSB module strengthens cross-scale feature capture through multi-scale receptive field fusion; the OA module precisely focuses on target regions via fine-grained attention within local windows; and the B-SiLU activation function optimizes gradient flow and feature discrimination through improved nonlinear mapping. These modules work in concert and are integrated into the YOLOv11 model, significantly boosting its performance in photovoltaic fault detection and raising the detection accuracy to 81.6%. The heatmap visualizations further verify the effectiveness of these improvements, providing strong support for future research and applications.

## 5. Conclusions and Future Work

The YOLOv11-MO framework proposed in this paper integrates MSB and OA modules, demonstrating significant improvements in detecting photovoltaic faults across various categories, including Clean, Damaged, Dirty, Panel, and Snow. The integration of these modules has enhanced the model’s ability to handle complex backgrounds and small objects. Specifically, the MSB has proven effective in capturing multi-scale features, while the OA module has bolstered the model’s attentional mechanisms toward key regions within images. These enhancements have collectively contributed to a more robust and accurate detection system. When compared to other YOLO models and RT-DETR, YOLOv11-MO achieves the highest detection accuracy of 0.816. This represents a notable improvement over the baseline model, with detection accuracies for Stain, Defect, and Snow increasing by 3.3%, 2.2%, and 0.8%, respectively. Despite these advancements, the increased computational complexity associated with the MSB module remains a trade-off that must be carefully managed. The model size has expanded from 18.3 MB to 59.3 MB, necessitating a balance between performance gains and resource consumption. This trade-off underscores the importance of tailored module selection based on specific application requirements. It is worth noting that, although the YOLOv11-MO framework has achieved remarkable progress in improving detection accuracy, the issue of false positives remains a challenge that requires attention. In complex photovoltaic scenarios, such as those involving shadows, reflections, or partial occlusions, the model may generate false positives. To address this issue, we have adopted a variety of strategies, including optimizing the training dataset of the model to increase its diversity and complexity, as well as adjusting the model’s parameters and improving the model structure to reduce the occurrence of false positives. Looking ahead, several promising research directions are worth exploring. First, it is crucial to develop more efficient module designs by leveraging advanced computational theories and optimization techniques. This will enable performance improvements while minimizing computational overhead. Second, integrating advanced multimodal data augmentation techniques, incorporating spectral and spatiotemporal information, can enhance the model’s robustness and ability to generalize across varied environmental settings. Third, exploring hybrid architectures that combine the strengths of different models through multidisciplinary approaches can lead to synergistic improvements in detection accuracy and efficiency. Finally, extending the application of these advancements to other domains, such as natural language processing and time-series analysis, could reveal new opportunities and validate the framework’s versatility and scalability. In summary, while the YOLOv11-MO framework has made substantial strides in PV fault detection, ongoing efforts to refine and extend its capabilities will be crucial in addressing the evolving challenges of this field.

## Figures and Tables

**Table 1:** Specifications of the DJI Mavic 3T thermal imaging drone.

Classification	Specification	Data Information
Fundamental Parameters	Dimensions (Deployed)	347.5 mm × 283 mm × 107.7 mm
	Operating Temperature Range	10 °C~40 °C
Parameters of Wide-Angle Camera	Image Resolution	4000 × 3000
	Sensor Type	CMOS
Parameters of Infrared Camera	Image Resolution	640 × 512
	Thermal Measurement Accuracy	±2 °C

**Table 2:** Distribution of classes and partitioning of the multimodal photovoltaic fault detection dataset.

Split	Total Images	Infrared Images	RGB Images	Requires Human Intervention			Does Not Require Human Intervention	
				Stain (Bird-Dropping)	Defect (Hot-Spot)	Snow	Panel	Clean
Training	5072	492	4580	12,736 (5239)	2991 (1132)	1372	1493	2760
Validation	612	42	570	2367 (486)	312 (101)	133	144	562
Test	444	24	420	1176 (238)	210 (56)	85	92	432
Overall	6128	558	5570	16,279 (5963)	3513 (1289)	1590	1729	3754

**Table 3:** Comparative experimental outcomes.

Model	mAP@0.5	Recall	Precision
YOLOv5 [26]	0.751	0.712	0.788
YOLOv8 [27]	0.772	0.737	0.803
YOLOv10 [28]	0.707	0.649	0.765
YOLOv11 [25]	0.798	0.771	0.822
YOLOv12 [29]	0.727	0.672	0.782
RT-DETR [22]	0.702	0.686	0.747

**Table 4:** Category-wise performance comparison of different models.

Map@0.5	YOLOv5	YOLOv8	YOLOv10	YOLOv11	YOLOv12	RT-DETR
Stain	0.613	0.608	0.574	0.615	0.597	0.603
Defect	0.641	0.707	0.567	0.813	0.601	0.554
Snow	0.792	0.821	0.739	0.833	0.752	0.755
Panel	0.761	0.774	0.718	0.766	0.737	0.674
Clean	0.949	0.953	0.937	0.965	0.951	0.928

**Table 5:** Ablation experimental outcomes.

YOLOv11	HSV	OA	MSB	B-SiLU	mAP@0.5	Recall	Precision	Parameters (MB)	FPS
(i)	×	×	×	×	0.798	0.771	0.822	18.3	17.441
(ii)	√	×	×	×	0.799	0.762	0.821	18.3	18.398
(iii)	×	√	×	×	0.801	0.766	0.814	18.3	18.320
(iv)	×	×	√	×	0.804	0.77	0.817	59.3	14.282
(v)	√	√	×	×	0.808	0.791	0.818	18.3	18.349
(vi)	√	×	√	×	0.809	0.779	0.820	59.3	14.593
(vii)	×	√	√	×	0.811	0.791	0.822	59.3	14.522
(viii)	√	√	√	×	0.812	0.786	0.824	59.3	14.619
(ix)	√	√	√	√	0.816	0.773	0.835	59.3	14.637

**Table 6:** Performance comparison of mAP@0.5 for different target categories with various module combinations.

Map@0.5	(i)	(ii)	(iii)	(iv)	(v)	(vi)	(vii)	(viii)	(ix)
Stain	0.615	0.643	0.63	0.639	0.638	0.634	0.651	0.631	0.648
Defect	0.813	0.779	0.812	0.789	0.837	0.823	0.812	0.809	0.835
Snow	0.833	0.84	0.832	0.828	0.842	0.848	0.851	0.853	0.841
Panel	0.766	0.775	0.758	0.811	0.757	0.78	0.791	0.811	0.808
Clean	0.965	0.955	0.956	0.957	0.960	0.961	0.949	0.955	0.949

**Table 7:** Comparison of detection performance across different types of photovoltaic modules.

Photovoltaic Module Type	Dataset Size	mAP@0.5	Recall	Precision
Monocrystalline Silicon	6128	0.816	0.773	0.835
Polycrystalline Silicon	2884	0.867	0.838	0.836
Thin-Film	203	0.723	0.721	0.671
Bifacial	182	0.737	0.833	0.664

## Data Availability

The datasets used and analyzed in the current study are available from the corresponding author upon reasonable request.
